# Improved Drought Tolerance by AMF Inoculation in Maize (*Zea mays*) Involves Physiological and Biochemical Implications

**DOI:** 10.3390/plants8120579

**Published:** 2019-12-06

**Authors:** Naheeda Begum, Muhammad Abass Ahanger, Yunyun Su, Yafang Lei, Nabil Sabet A. Mustafa, Parvaiz Ahmad, Lixin Zhang

**Affiliations:** 1 College of Life Sciences, Northwest A&F University, Yangling 712100, Shaanxi, China; naheedabot2@nwafu.edu.cn (N.B.); ahangerma@gmail.com (M.A.A.); suyunyun1991@163.com (Y.S.); leiyafang0917@163.com (Y.L.); 2 Biotechnology for fruit Tress Micropropagation Laboratory, Department of Pomology, National Research Centre, Cairo 12622, Egypt; nabilhotline@yahoo.com; 3 Botany and Microbiology Department, College of Science, King Saudi University, Riyadh 11451, Saudi Arabia; parvaizbot@yahoo.com

**Keywords:** antioxidants, oxidative damage, *Zea mays*, drought stress, *Glomus versiforme*

## Abstract

The role of arbuscular mycorrhizal fungus (AMF, *Glomus versiforme*) in amelioration of drought-induced effects on growth and physio-biochemical attributes in maize (*Zea mays* L.) was studied. Maize plants were exposed to two drought regimes, i.e., moderate drought (MD) and severe drought (SD), with and without AMF inoculation. Drought at both levels reduced plant height, and chlorophyll and carotenoid content, thereby impeding photosynthesis. In addition, drought stress enhanced the generation of toxic reactive oxygen species (ROS), including H_2_O_2_, resulting in membrane damage reflected as increased electrolyte leakage and lipid peroxidation. Such negative effects were much more apparent under SD conditions that those of MD and the control, however, AMF inoculation significantly ameliorated the deleterious effects of drought-induced oxidative damage. Under control conditions, inoculation of AMF increased growth and photosynthesis by significantly improving chlorophyll content, mineral uptake and assimilation. AMF inoculation increased the content of compatible solutes, such as proline, sugars and free amino acids, assisting in maintaining the relative water content. Up-regulation of the antioxidant system was obvious in AMF-inoculated plants, thereby mediating quick alleviation of oxidative effects of drought through elimination of ROS. In addition, AMF mediated up-regulation of the antioxidant system contributed to maintenance of redox homeostasis, leading to protection of major metabolic pathways, including photosynthesis, as observed in the present study. Total phenols increased due to AMF inoculation under both MD and SD conditions. The present study advocates the beneficial role of *G. versiforme* inoculation in maize against drought stress.

## 1. Introduction

Environmental stress factors adversely influence plant growth and development. Among the key abiotic environmental factors, drought stress is one of the most severe constraints to crop productivity. Damaging effects of drought on crop production depend on the severity and duration of the stressful conditions [1]. Drought stress more often occurs in the areas of arid and semi-arid climatic regions around the globe [2], thereby imposing a significant threat to world food security. Reduced availability of water has been reported to reduce crop growth and yield by influencing enzyme activity, mineral uptake and assimilation [1,3]. For avoiding drought stress, plants cut water losses by reducing transpiration and developing deep root systems to improve water uptake [4]. Drought-induced decline in photosynthesis results from turgor loss with limited access to CO_2_, resulting from stomatal closure and damage to photosystem I (PSI) and PSII components of photosynthetic apparatus [5]. Among the key determinants of growth reductions due to drought are considered the excess accumulation of reactive oxygen species (ROS), which considerably damage structural and functional integrity of cellular organelles and the whole plant [6,7]. Plants have evolved key mechanisms to relieve the harmful effects of excess accumulated ROS to protect the stability of cellular metabolism and improve yield productivity [7,8]. The key mechanisms that attenuate the deleterious effects include: (a) improved osmotic regulation, (b) antioxidant system and (c) secondary metabolite accumulation [6]. The antioxidant system is constituted of enzymatic and non-enzymatic components, which coordinate for the protection of the plant metabolism by eliminating the excess ROS; however, osmolytes, including proline, glycine betaine, and sugars, function to maintain the tissue water content. All these protective mechanisms result in protection of structural and functional aspects of macromolecules, including proteins, enzymes, and nucleic acids [6]. Considerable evidence is available indicating the alleviation of stress-induced deleterious effects due to up-regulation of the antioxidant system, and increased accumulation of osmolyte and secondary metabolites [7,9].

Arbuscular mycorrhizal fungi (AMF) include widely spread species of endotrophic fungi occurring in almost all terrestrial ecosystems and have been reported to form symbiotic associations with flowering plants, ferns and bryophytes [10,11]. Beneficial soil microbes, including AMF, affect plant growth by maintaining the soil health, as well as modifying the root architect. Improved soil moisture and other properties due to AMF have been reported to enhance the growth capacity and impart drought tolerance of plants [12,13]. AMF have been reported to influence the stress tolerance mechanisms significantly leading to optimization of biochemical changes arising due to physiological modification, osmoregulation, etc. [14,15,16]. AMF mediated enhancement of growth results from the significant improvement in soil structure and mineral status [17,18]. Due to their potential to stimulate growth under extreme conditions, AMF are considered as bio-ameliorators of stress, resulting in protection of major cellular pathways [14,19]. Modulations in the morphological and physio-biochemical attributes, such as mineral assimilation and enzyme activity, due to AMF are reflected in improved yield, biomass accumulation and vigor [14,20]. AMF up-regulate the gene expression of stress responsive genes [21] and also enhance the synthesis of key molecules, such as metalothioniens and polyamines [22], for counteracting the damaging effects of stresses. Plant species colonized with AMF display enhanced growth and resistance to drought [16]. AMF colonized plants exhibit greater chlorophyll content and photosynthetic functioning [23,24,25]. The metabolic and physiological attributes of mycorrhiza results in better growth under drought conditions, as well as hydration of tissues and stomatal conductance, improved photosynthetic efficiency and osmotic adjustment, and mitigation of oxidative stress [26,27,28,29].

Maize (*Zea mays* L.) is known as corn and is part of the family Poaceae. It is one of the most widely grown grain crops, consumed as a staple food by millions of people across the globe. It is an essential component of global food security utilized by human beings, and an important component for the production of corn ethanol, animal fodder, corn starch and corn syrup. However, its growth is significantly affected by stress factors such as drought, though it is grown widely in tropical areas of China. Against this backdrop, it was hypothesized that inoculation of AMF (*G. versiforme*) can protect growth and photosynthesis of maize by up-regulating its tolerance mechanisms, including the antioxidant system, osmolyte and secondary metabolite accumulation, and mineral nutrition.

## 2. Materials and Methods

### 2.1. Experimental Materials and Treatment

Experiments were carried at the College of Life Science, Northwest A&F University, Yangling, Shaanxi, China. The top layer (0–20 cm) of soil having pH 7.5, water field capacity of 52.21%, and N, P and K concentrations of 65.98, 18.78 and 80.67 mg kg^−1^ soil, respectively, was taken from the campus of Northwest A&F University (Yangling, Shaanxi Province, China). Soil was sterilized at 121 °C for two hours, and sterilized soil was used to fill pots (20 cm diameter). To half of the pots 10 g of AMF (*G. versiforme*) inoculum in the form of maize root fragments was added five days before seed sowing. The AMF (*G. versiforme*) inoculum, which included infected maize roots and spores, was obtained from the Horticulture Department, Northwest A&F University, Yangling, Shaanxi, P.R. China. The obtained inoculum was replicated for two consecutive years in a glasshouse with an average day/night temperature of 32/27 °C with a normal relative humidity [30].

Healthy seeds of maize (*Zea mays* L.) were surface sterilized with 1% sodium hypochlorite solution and washed by distilled water. Pots were regularly monitored and irrigated, however, half-strength Hoagland solution (100 mL pot^−1^) was given once a week to prevent nutrient deficiency. Twenty days after seedling establishment, the number of seedlings per pot was thinned to three and these were exposed to drought stress for another 30 days, maintaining the stress intensity as moderate and severe drought. Pots were maintained in a growth room with a day/night temperature of 33/28 °C and relative humidity of 65 ± 5.0% in a completely randomized (CR) design with three replicates for each treatment. Details of treatments were as follows: (a) control, (b) moderate drought (MD), (c) severe drought (SD), (d) AMF, (e) MD + AMF and (f) SD + AMF. After 30 days of moderate and severe drought, plants were carefully uprooted and taken to the laboratory for experimentation. Root tissues were fixed in formalin acetic acid alcohol until further use.

### 2.2. Measurement of Growth Parameters

Plant height was measured using a manual scale and the dry weight of shoots was measured after drying the tissues in an oven for 48 h at 70 °C.

### 2.3. Root Colonization

The fixed roots were cleaned and cut into small pieces (2 cm) in length and treated with 5% KOH for 30 min at 90 °C. According to Phillips and Hayman, [31] the plants roots were stained with 0.05% trypan blue. Stained root tissues were examined under light microscope model (Olympus, Japan). Similarly, the percentage of colonization was recorded following the technique of Road and Division, [32] with at least 50 root segments per treatment using the following formula:

Root colonization (%) = Number of colonized roots segments/Number of observed roots segments × 100,(1)

### 2.4. Relative Water Content Measurements

The measurements of relative water content (RWC) in fresh leaves was done following the method outlined by Weatherley [33]. Briefly, leaf discs were punched from fresh leaves and weighed (FW). The same discs were allowed to gain turgidity by floating them in petri dishes containing distilled water and their turgid weight (TW) was recorded. After oven drying, the discs’ dry weight (DW) was taken and calculation of RWC was done using the following formula:

RWC (%) = FW − DW/TW − DW × 100,(2)

### 2.5. Photosynthetic Pigments Determination and Gas Exchange Parameters

Determination of photosynthetic pigments (chlorophyll a, b, total chlorophylls and carotenoids) was done using the method of Arnon [34]. Using acetone (80%, v/v) the fresh leaves were homogenized and the supernatant was read at 663, 645 and 480 nm.

Gas exchange parameters, including photosynthesis (Pn), intercellular CO_2_ concentration (Ci), transpiration (E), and stomatal conductance (gs), were measured using the portable photosynthesis system LI-COR-6400. Measurement of maximal photochemical efficiency (Fv/Fm), photochemical quenching, non-photochemical quenching and the electron transport rate in drought and AMF-treated plants was conducted using a Modulated Chlorophyll Fluorometer (PAM 2500; Walz, Germany) after dark adaptation of leaves for 25 min.

### 2.6. Estimation of Proline, Glycine Betaine, Free Amino Acids and Sugar

For estimation of free proline, plant tissue was macerated in sulphosalicylic acid at 3% using pestle and mortar. After centrifugation at 3000× *g* for 10 min, supernatant (2 mL) was mixed with 2 mL each of glacial acetic acid and ninhydrin reagent followed by incubation at 100 °C for 1 h. On an ice bath, after terminating the reaction, toluene was used for separation of the proline while optical density was taken at 520 nm [35].

For estimation of glycine betaine, 500 mg dry sample was extracted in 20 mL distilled water by shaking overnight at room temperature. After filtration, a 0.5 mL aliquot was mixed with 0.2 mL cold potassium iodide, and periodide crystals were dissolved in 1,2-dichloroethane and kept for 3 hrs. Afterward, we noted its absorbance at 365 nm and calculation was carried out from the standard curve [36].

The content of free sugars was estimated by adopting the anthrone method. Briefly, a 100 mg dry powdered sample was extracted in 80% ethanol and homogenate was centrifuged at 5000× *g* for 10 min. Supernatant was made up to 1 mL, and 4 mL anthrone (0.2%) was added and optical density was measured at 620 nm [37,38].

The protocol outlined in [39] as described by [40] was followed for estimation of free amino acids. After extraction in 80% ethanol, 100 µL supernatant was incubated with 1 mL ninhydrin reagent for 30 min and optical density was recorded at 570 nm.

### 2.7. Estimation of Total Phenols

Phenols were extracted by homogenizing a 500 mg dry sample in 80% ethanol. After centrifugation for 10 min at 10000× *g*, 0.1 mL supernatant was made to 2 mL using distilled water. Thereafter, 1 N Folin–Ciocalteu reagent and Na_2_CO_3_ were added and absorbance was read at 765 nm [41]. Calculation was done from the standard curve of gallic acid.

### 2.8. Assay of Antioxidant Enzymes

Antioxidant enzymes were extracted by macerating fresh plant tissue in chilled 100 mM (pH 7.8) phosphate buffer with polyvinyle pyrrolidine (PVP, 0.1%) and ethylenediaminetetraacetic acid (EDTA, 0.5 mM) using a pre-chilled pestle and mortar. After centrifuging the homogenate at 12000× *g* for 15 min at 4 °C, the supernatant was used for enzyme assay. However, for the extraction of ascorbate peroxidase (APX), the extraction buffer was supplemented with 2 mM ascorbate.

For assaying superoxide dismutase (SOD) activity, the ability of the enzyme to inhibit the photochemical reduction of nitroblue tetrazolium (NBT) was recorded at 560 nm according to the method of Dhindsa et al. [42]. The content of protein causing 50% photo-reduction was considered as 1 unit of enzyme activity and expressed as enzyme unit (EU) mg^−1^ protein. Catalase (CAT) activity was assayed by monitoring the reduction in optical density at 240 nm for 2 min using H_2_O_2_ as substrate. Activity was calculated using extinction coefficient (ε) of 0.036 mM^−1^ cm^−1^ and expressed as EU mg^−1^ protein [43]. The method of Nakano and Asada [44] was used to assay the activity of ascorbate peroxidase (APX), and H_2_O_2_-dependent oxidation of ascorbate was examined spectrophotometrically at 290 nm. An extinction coefficient (ε) of 2.8 mM^−1^ cm^−1^was used for calculation and activity was expressed as EU mg^−1^ protein. Activity of glutathione reductase (GR) was measured following the method of Foyer and Halliwell [45]. Glutathione-dependent oxidation of nicotinamide adenine dinucleotide phosphate (NADPH) was recorded at 340 nm. An extinction coefficient of 6.2 mM^−1^ cm^−1^ was used for calculation and activity was expressed as EU mg^−1^ protein. Activity of peroxidase (POD) was assayed according to the method of [46] in a reaction mixture containing enzyme extract, phosphate buffer (50 mM, pH 7.0), 1 mM guaiacol and 1 mM H_2_O_2_. Change in absorbance was noted at 470 nm for 3 min.

### 2.9. Estimation of Reduced Glutathione and Ascorbic Acid

Reduced glutathione (GSH) content was estimated following Moron, et al. [47]. Extracts were reacted with 5’ 5’- dithiobis-2-nitrobenzoic acid (DTNB) and optical density was taken at 412 nm. Content of ascorbic acid was estimated by adopting the methodology designated by Omayl et al. [48]. Computations were done using standard curves of AsA and GSH for assessment of AsA and GSH, respectively.

### 2.10. Determination of Hydrogen Peroxide (H_2_O_2_) and Lipid Peroxidation

Content of hydrogen peroxide (H_2_O_2_) was estimated spectrophotometrically following the method described by [49]. Absorbance was measured at 390 nm and concentration of H_2_O_2_ was calculated from the standard curve of H_2_O_2_.

For measurement of lipid peroxidation, fresh leaves were homogenized in 0.1% trichloroacetic acid (TCA). After centrifugation, supernatant was reacted with 0.5% thiobarbituric acid (TBA, prepared in 20% TCA) at 95 °C for 30 min. Absorbance was read at 532 and 600 nm and an extinction coefficient (ε) of 155 mM^−1^ cm^−1^ was used for calculation [50].

### 2.11. Nitrate Reductase Determination

In order to assess the activity of nitrate reductase (NR), fresh leaves were incubated in potassium phosphate buffer (100 mM, pH 7.5) containing 200 mM KNO_3_ and 0.5% n-propanol (v/v) for three hours in dark conditions at 30 °C. Thereafter, 1 mL aliquot was added with an equal volume of sulphanilamide (1%) and 1-nephthylethylene diamine dihydrochloride (0.2%) and kept for 20 min. Optical density was taken at 540 nm [51].

### 2.12. Determination of Tissue Nitrogen (N), Phosphorous (P) and Potassium (K)

Nitrogen was determined by a modified micro-Kjeldahl method [52]. Potassium was measured using a flame photometer connected with continuous flow systems (microflow automated continuous-flow analyzer III, Italy). A spectrophotometer was used to determine the phosphorous [53].

### 2.13. Statistical Analysis

Results are means (± SE) of three replicates. Statistical analysis was carried out using SPSS 17 and significant differences at *p* < 0.05 were determined.

## 3. Results

### 3.1. Effect of Drought and AMF on Growth, Biomass and Colonization

Drought stress at both levels reduced growth in terms of height and dry weight, and AMF inoculation resulted in significant amelioration of the decline. Relative to control, plant height and dry weight declined maximally by 40.66% and 53.25% due to SD. However, AMF-inoculated plants exhibited significant increase in height (36.32%) and dry weight (75.73%) over the control. Similarly, inoculation of AMF to drought-stressed seedlings ameliorated the decline by 52.09% and 42.51% for height, and 62.45% and 29.74% for dry weight, over the moderately stressed (MD) and severely stressed (SD) seedlings, respectively. Both drought levels significantly reduced the AMF colonization and a maximal decline of 38.09% was observed in severely stressed (SD) plants (Table 1).

### 3.2. Effect of Drought and AMF on Photosynthesis and Chlorophyll Content

**Figure 1:**
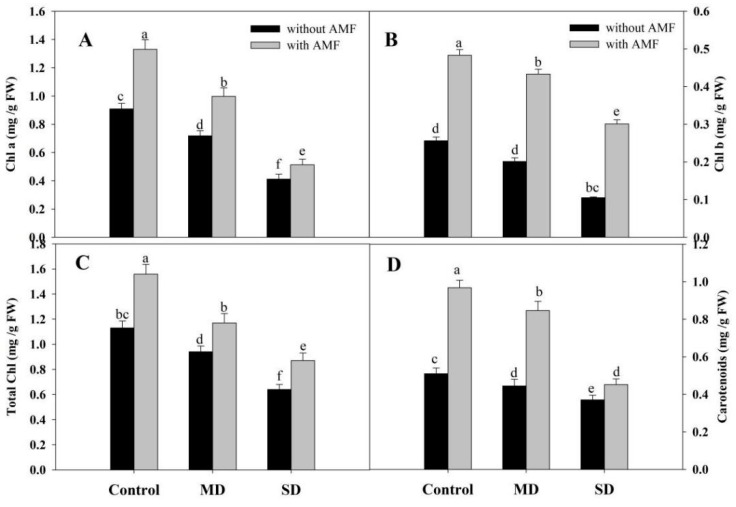
Effect of drought stress on (**A**) chlorophyll a (Chl a), (**B**) Chl b, (**C**) total chlorophylls and (**D**) carotenoids in *Zea mays* L. with and without AMF inoculation. Data presented are means (± SE) of three replicates and different letters show significant difference at *p* < 0.05. MD—moderate drought, SD—severe drought, AMF—*G. versiforme*.

**Figure 2:**
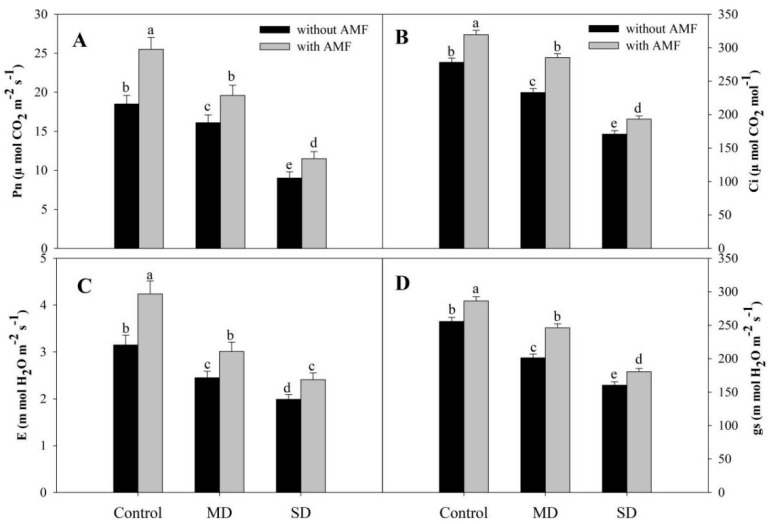
Effect of drought stress on (**A**) photosynthesis, (**B**) stomatal conductance, (**C**) intercellular CO_2_ and (**D**) transpiration in *Zea mays* with and without AMF inoculation. Data presented are means (± SE) of three replicates and different letters show significant difference at *p* < 0.05. MD—moderate drought, SD—severe drought, AMF—*G. versiforme*.

**Figure 3:**
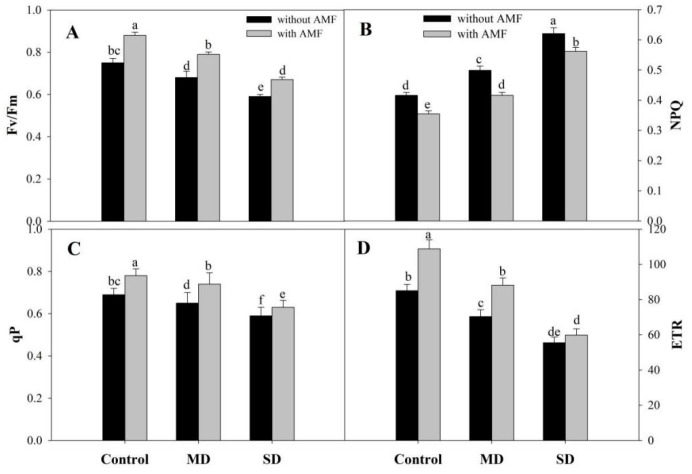
Effect of drought stress on (**A**) PSII efficiency (Fv/Fm) (**B**) photochemical quenching (qP), (**C**) non-photochemical quenching (NPQ) and (**D**) electron transport rate in *Zea mays* L. with and without AMF inoculation. Data presented are means (± SE) of three replicates and different letters show significant difference at *p* < 0.05. MD—moderate drought, SD—severe drought, AMF—*G. versiforme*.

Drought reduced chlorophyll (Chl) a, b, total Chl and carotenoid content by 54.67%, 64.52%, 43.36% and 40.98% respectively under SD. However, AMF inoculation significantly enhanced the content of pigments with percentage increases of 45.94% for Chl a, 88.67% for Chl b, 38.05% for total Chl, and 89.80% for carotenoids compared to the control. At both drought levels, AMF inoculation significantly ameliorated the decline over the non-inoculated counterparts. An amelioration of 25.62% for Chl a, 115.4% for Chl b, 24.46% for total Chl, and 90.11% for carotenoid was observed due to AMF inoculation under MD conditions (Figure 1A–D). Exposure to SD reduced Pn by 51.29%, Ci by 38.60%, E by 36.82%, and gs by 37.20% compared to the control, however, increases of 37.83%, 14.77%, 34.60% and 12.01% in Pn, Ci, E and gs, respectively, were observed in AMF-inoculated plants. AMF inoculation mitigated the drought-induced decline to a considerable extent, imparting 27.63%, 13.17%, 21.10% and 12.46% amelioration in Pn, Ci, E and gs, respectively, in SD + AMF-treated plants compared to SD-stressed plants (Figure 2A–D). Drought reduced the fluorescence parameters, including efficiency of PSII (Fv/Fm), photochemical quenching (qP), and electron transport rate (ETR), while increasing non-photochemical quenching (NPQ) significantly compared to the control plants. Inoculation of AMF increased Fv/Fm by 17.33%, qP by 13.04% and ETR by 27.84%, while reducing NPQ by 14.66% compared to the control plants. Inoculation of AMF increased Fv/Fm, qP and ETR by 16.17%, 13.84% and 25.28% in MD + AMF-treated plants, and by 13.55%, 6.77% and 7.92% in SD + AMF-treated plants, compared to the respective MD and SD counterparts. Drought-induced increase in NPQ was also reduced by 16.63% and 9.50% in MD + AMF and SD + AMF over their respective drought-stressed plants (Figure 3A–D).

### 3.3. Effects of AMF on Lipid Peroxidation and Hydrogen Peroxide

**Figure 4:**
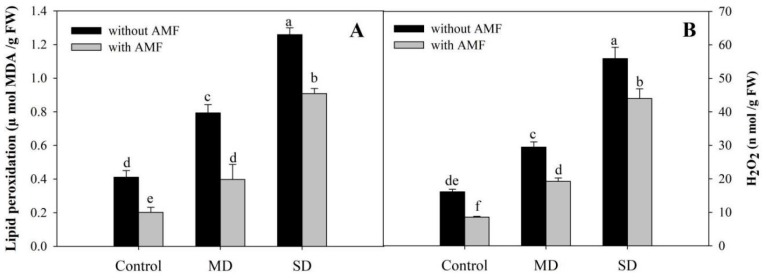
Effect of drought stress on (**A**) lipid peroxidation and (**B**) hydrogen peroxide in *Zea mays* L. with and without AMF inoculation. Data presented are means (± SE) of three replicates and different letters show significant difference at *p* < 0.05. MD—moderate drought, SD—severe drought, AMF—*G. versiforme*.

Results depicting the influence of AMF and drought stress on H_2_O_2_ and lipid peroxidation are shown in Figure 4. Relative to the control, H_2_O_2_ concentration increased by 83.22% and 247.20% due to exposure to MD and SD, thereby resulting in enhancements of 92.94% and 206.32%, respectively, in lipid peroxidation. However, AMF-inoculated plants showed 46.83% and 50.85% declines in H_2_O_2_ and lipid peroxidation, respectively, compared to the uninoculated control. AMF inoculation reduced the accumulation of H_2_O_2_ under drought stress, thereby reducing the rate of lipid peroxidation. In comparison to plants exposed to MD and SD, H_2_O_2_ and lipid peroxidation declined significantly in MD + AMF and SD + AMF, respectively (Figure 4A,B).

### 3.4. Effect of AMF on Activities of Antioxidant Enzymes

**Figure 5:**
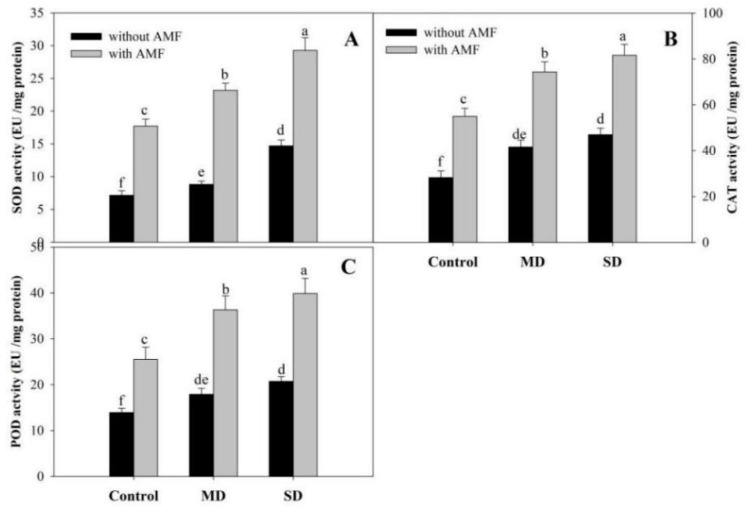
Effect of drought stress on activity of (**A**) superoxide dismutase, (**B**) catalase, and (**C**) peroxidase in *Zea mays* L. with and without AMF inoculation. Data presented are means (± SE) of three replicates and different letters show significant difference at *p* < 0.05. MD—moderate drought, SD—severe drought, AMF—*G. versiforme*.

**Figure 6:**
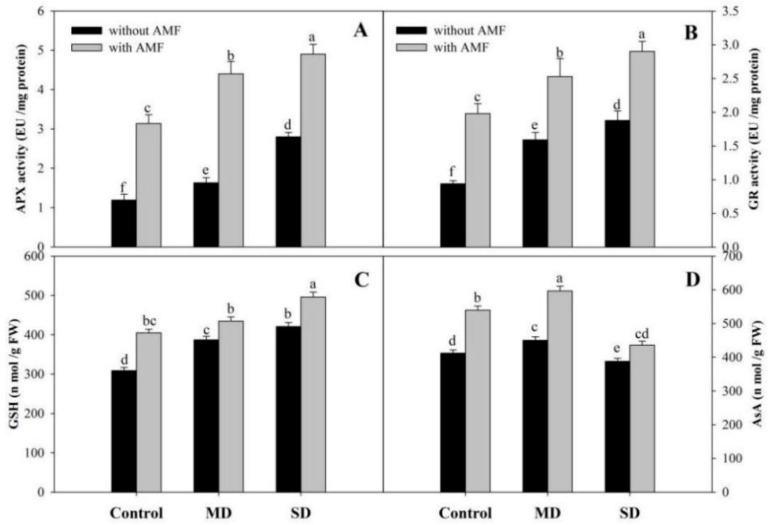
Effect of drought stress on activity of (**A**) ascorbate peroxidase, (**B**) glutathione reductase, and content of (**C**) reduced glutathione and (**D**) ascorbic acid in *Zea mays* L. with and without AMF inoculation. Data presented are means (± SE) of three replicates and different letters show significant difference at *p* < 0.05. MD—moderate drought, SD—severe drought, AMF—*G. versiforme*.

Activities of assayed antioxidant enzymes registered an increase due to drought-stress exposure, however, AMF inoculation significantly increased the activities of antioxidant enzymes under normal as well as drought conditions over the uninoculated plants (Figure 5 and Figure 6). Relative to the control, the percentage increases in activities of SOD, CAT, POD, APX and GR were 23.21%, 8.61%, 28.49%, 36.97% and 69.14%, respectively, due to exposure to MD. Relative to SD, AMF further enhanced the activities of SOD by 98.63%, CAT by 73.61%, POD by 92.47%, APX by 75.00% and GR by 54.22%, depicting the beneficial role of AMF in improving drought tolerance. Under control conditions, AMF inoculation resulted in significant increases in the activities of SOD, CAT, POD, APX and GR by 2.4-, 1.4-, 1.8-, 2.6- and 2.1-fold, respectively, compared to the control (Figure 5A–C; Figure 6A,B).

Content of GSH and AsA increased significantly due to AMF inoculation compared to the control. Plants exposed to MD and SD exhibited increases of 25.24% and 36.24% in GSH accumulation compared to the control, however, AsA content increased by 9.19% due to MD, while a decline of 5.84% was observed due to SD. Inoculation of AMF further enhanced the accumulation of GSH and AsA, and ameliorated the decline in AsA by 12.36% in SD + AMF-treated plants compared to the SD (Figure 6C,D).

### 3.5. Effects of AMF and Drought on RWC and Osmolyte Content

**Figure 7:**
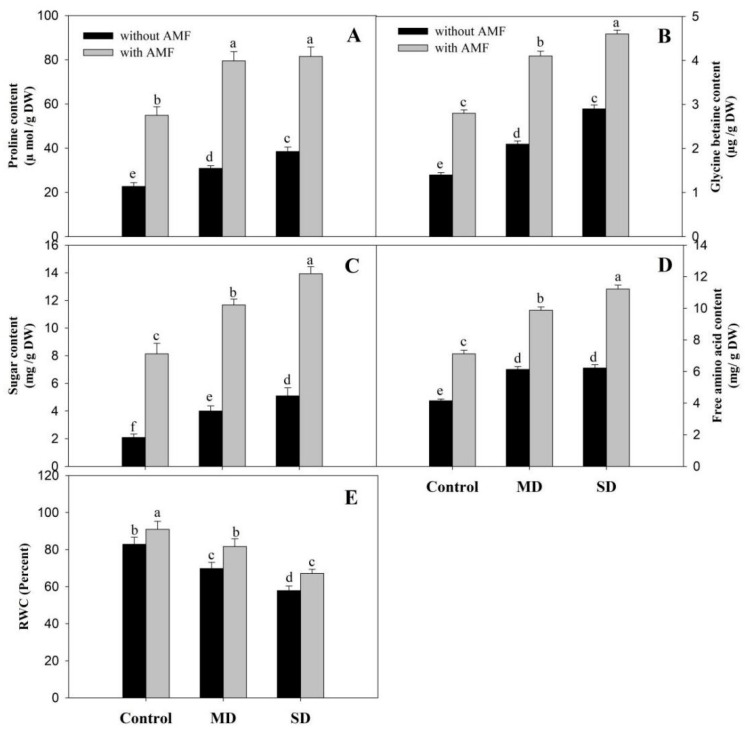
Effect of drought stress on (**A**) proline, (**B**) glycine betaine, (**C**) sugars, (**D**) free amino acids and (**E**) relative water content in *Zea mays* L. with and without AMF inoculation. Data presented are means (± SE) of three replicates and different letters show significant difference at *p* < 0.05. MD—moderate drought, SD—severe drought, AMF—*G. versiforme*.

Results showing the effect of AMF and drought on the accumulation of proline, free amino acids, glycine betaine and sugars are shown in Figure 7. Drought stress at both levels resulted in increased accumulation of proline, free amino acids, glycine betaine and sugars compared to the control, with maximal increases of 69.84%, 49.87%, 107.14% and 142.8%, respectively, due to SD exposure. Inoculation of AMF proved effective in improving the contents of proline, free amino acids, glycine betaine and sugars, by 2.4-, 1.7-, 2.0- and 3.8-fold, respectively, over the uninoculated control. Such an effect was maintained under drought-stress conditions and maximal increases of 259.34% for proline, 170.36% for free amino acids, 228.57% for glycine betaine and 563.33% for sugars, compared to the control plants was attained in SD + AMF (Figure 7A–D). RWC declined at both drought levels with maximal reduction of 30.12% due to SD. However, AMF inoculation increased RWC by 9.80% and ameliorated the decline by 17.02% in MD + AMF and 16.06% in SD + AMF, compared to their respective drought-stressed counterparts (Figure 7E).

### 3.6. Effect of AMF and Drought on Total Phenols

**Figure 8:**
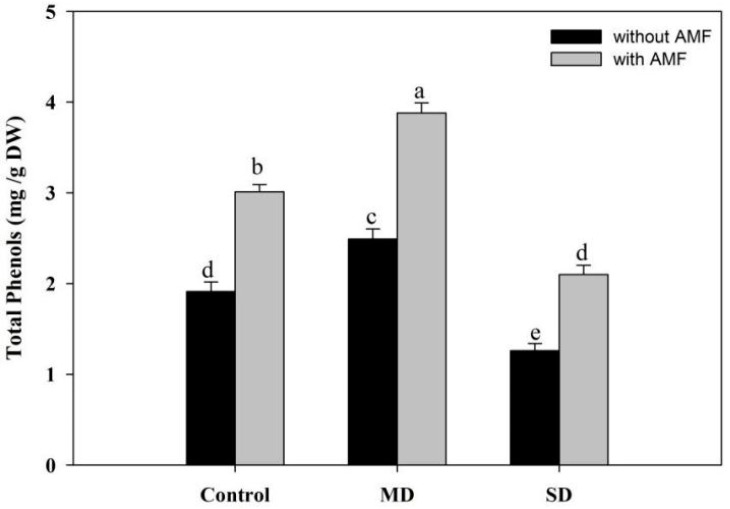
Effect of drought stress on total phenols in *Zea mays* L. with and without AMF inoculation. Data presented are means (± SE) of three replicates and different letters show significant difference at *p* < 0.05. MD—moderate drought, SD—severe drought, AMF—*G. versiforme*.

Phenols increased significantly due to MD (28.79%) exposure while declining by 34.03% due to SD over the control. Inoculation of AMF increased the accumulation of phenols by 57.59% compared to the control and caused further enhancement when used to inoculate to drought-stressed plants. Under MD stress conditions, AMF further increased phenol content, while completely ameliorating the decline induced due to SD (Figure 8).

### 3.7. Effect of AMF on Activity of NR and Content of Nitrogen, Phosphorous and Potassium

Activity of NR was reduced significantly due to drought stress, with a 70.69% decline due to SD exposure. Significant enhancement was observed due to AMF inoculation compared to the control. Inoculation of AMF to drought-stressed plants increased the activity of NR by 138.6% and 122.3% in MD + AMF and SD + AMF over the MD and SD stressed plants, respectively. Content of N, P and K were reduced significantly due to drought stress, attaining maximal declines of 49.62%, 54.71% and 42.05% due to SD; however, under control growth conditions, AMF inoculation significantly increased the content of N, P and K by 37.88%, 60.00% and 62.80%, respectively, compared to the control. Significant amelioration of drought-induced decline was apparent due to AMF inoculation with the impact being more obvious under MD conditions. Relative to MD stressed plants, amelioration of 30.30%, 38.55% and 68.55% was observed in N, P and K, respectively, in MD + AMF-treated plants (Table 2).

## 4. Discussion

Drought stress has emerged as an alarming stress factor globally, and efficient strategies need to be devised so that excessive loss of agriculture production can be prevented. During the last decade, researchers have focused on implementing and validating many tools and techniques for preventing drought-mediated yield and productivity loss. In the present study we have investigated the influence of AMF species, *G. versiforme,* in improving the drought tolerance mechanisms in maize. Inoculation of maize with AMF significantly improved the growth and ameliorated the decline to a considerable extent. AMF-mediated growth increases of plants under drought stress has also been reported by other researchers [28,54,55,56,57,58,59]. Reduced water availability has been reported to affect the cellular division and proliferation by reducing the expression of key genes, such as tubulin and cyclin [60]. Recently, [61] also demonstrated significant amelioration of drought-induced declines in growth, height and biomass accumulation in *Zenia insignis* due to AMF inoculation. In the present study, drought at both levels reduced the colonization potential of AMF, which can be attributed to reduced availability of soil moisture. Similar to our results, reduced colonization under drought conditions has been reported by [61,62]. Increased plant growth due to AMF colonization has been ascribed to maintenance of increased mineral availability for absorption and tissue water content [63]. In drought-stressed damask rose, [64] also reported significant increase in uptake of essential mineral elements due to AMF inoculation, reflected in improved growth and drought-stress tolerance. AMF modifies root system elements, such as morphology and diameter, and promotes development of a dense root system to improve plant functioning [65]. Under water- and nutrient-deficient conditions, plants increase root fineness, root/shoot ratio and root number, as well as the length of root hairs, to counteract the deleterious effects [66]. In the present study, AMF may have modified the root morphology resulting in improved uptake of mineral elements, including N, P and K, as observed.

Increased growth in AMF-inoculated maize plants is directly regulated by their photosynthetic functioning. Nevertheless, drought at both levels significantly reduced the pigment content and photosynthetic attributes with a more apparent decline due to SD, and AMF inoculation proved beneficial to prevent the photosynthetic inhibition. Improved pigment content, gas exchange and PSII activity in AMF-colonized maize positively influenced the photosynthetic rate over the drought-stressed and uninoculated plants. Similar to our results, reduced chlorophyll content and photosynthesis due to drought has been reported by others [67,68,69]. Reduced photosynthesis under water stress is due to reduction in the coupling factor and production of ATP [70]. Both stomatal and non-stomatal parameters contribute to photosynthetic regulation, and in the present study, AMF inoculation significantly improved both gas exchange and fluorescence parameters, resulting in significant increase in photosynthesis. AMF-mediated increases in photosynthetic efficiency can be due to increased N uptake, which may result in greater Rubisco synthesis. The authors of [64] also reported increased content of chlorophyll and rate of photosynthesis in damask rose under drought stress due to AMF inoculation. Stress conditions trigger reduction in photosynthesis by reducing Rubisco synthesis [71] and up-regulating chlorophyllase activity [72], and such deleterious effects are intensified by hampered mineral nutrition, membrane functioning and enzyme stability. In the present study, AMF-mediated enhancement in photosynthesis in maize is due to the resultant effects on the stomatal and non-stomatal parameters. Increased stomatal functioning enhances CO_2_ entry into the leaf tissues and its successful assimilation was observed in AMF-inoculated plants, reflected in terms of increased PSII activity; such results have also been reported in AMF-inoculated wheat [73]. Similar to our results, [74] also demonstrated increased photosynthetic performance of watermelon due to AMF inoculation. Stressful growth conditions increase NPQ due to excess accumulation of protons leading to saturation of the electron transport chain [75,76,77]. Plants tend to mitigate the effects of stresses at the chloroplast level by increasing NPQ so as to dissipate excess excitation energy [69,78]. AMF inoculation regulates Rubisco activity and prevents photoinhibition under drought stress, reflected in considerable amelioration of deleterious effects [74].

*Zea mays* exposed to drought stress showed an apparent decline in the activity of nitrate reductase and the uptake of N, with more reduction due to severe drought (SD). Inoculation of AMF significantly improved the activity of NR and also mitigated the drought-induced decline. Similar to our findings, a drought-induced decline in NR activity and N uptake has been earlier reported in *Triticum aestivum* L. [3] and *Vigna unguiculata* [79]. Increased N uptake and NR activity due to AMF inoculation may have contributed to increased synthesis of nitrogenous amino acids, thereby contributing to maintenance of several key biosynthetic pathways. NR catalyzes the rate-limiting step in nitrogen assimilation, and enhancement of its activity has been reported to directly influence the synthesis of key stress-protective amino acids, such as proline, thereby leading to photosynthetic regulation under stress [80]. In the present study, AMF mediated up-regulation of NR activity and N uptake under drought, justifying its potential benefit for maize drought tolerance. In addition, AMF improved uptake of K under normal as well as drought stress conditions. K is considered an important inorganic osmolyte involved in amelioration of deleterious effects of stresses on plants through modulation of the antioxidant system, and osmolyte and secondary metabolite accumulation [3]. AMF-inoculated plants showed increased phosphorus content compared to non-AMF-inoculated plants. Moreover, increased P concentration has also been reported in maize plants by [81] under drought stress. In addition, [82] were of the view that AMF inoculation improved P concentration in plants and enhanced drought stress tolerance. It is believed that increased P uptake due to AMF inoculation makes a significant contribution to the ecosystem, as well as increasing tolerance of plants against drought [83]. Earlier, increased uptake of minerals, including N, Mg and K, in AMF-inoculated cucumber was reported by [84]. AMF-mediated increases in mineral uptake may also significantly contribute to cell division and proliferation, which are prone to drought conditions. Exposure to drought conditions hampers mineral uptake by reducing their accessibility by roots [20].

AMF inoculation of drought-stressed maize plants imparted obvious declines in the oxidative damage to membranes by reducing the generation of H_2_O_2_. In plants, H_2_O_2_ generated at various sites in different organelles may diffuse through membranes, thereby causing damage over long distances. Increased membrane damage in terms of lipid peroxidation in *Brassica napus* due to excessive generation of ROS has been earlier demonstrated [85]. Drought influences plant growth transitions by causing a surge in H_2_O_2_ accumulation and impeding the membrane integrity [86]. Stresses up-regulate lipoxygenase activity [87] and alter the poly-unsaturated fatty acid composition [88], thereby resulting in loss of membrane structural and functional integrity; however, it can be inferred that AMF inoculation may maintain a higher poly-unsaturated fatty acid composition under stressed conditions, as has been reported in *Ephedra alata* [88]. AMF inoculation of maize exposed to two drought regimes maintained relatively low concentrations of H_2_O_2_, which can be attributed to up-regulation of their antioxidant metabolisms, thereby tending to optimize the ROS concentrations to minimal possible levels and protecting the major metabolic processes, such as photosynthesis. Maintaining low concentrations of ROS benefits plants in regulating and integrating key developmental events, including germination and root growth, cell proliferation, stomatal functioning, signaling, programmed cell death and senescence [89,90].

AMF inoculation resulted in significant enhancement in the activities of the antioxidant enzymes studied, making maize plants well equipped to counteract the deleterious effects of drought regimes. SOD mediated dismutation of superoxide radicals generated in the chloroplast prevents damage to the photosynthetic apparatus [91]. Increased SOD activity due to AMF prevents the generation of toxic hydroxyl radicals through the Haber–Weiss reaction, which can otherwise induce serious damage to membranes and organelle functioning. CAT and POD neutralize excess H_2_O_2_ in cytosol, while APX, GR, AsA and GSH act in an intriguing pathway (AsA–GSH) to neutralize H_2_O_2_ in the chloroplast and mitochondria [7]. Increased activities of antioxidant enzymes lead to elimination of excessive ROS, thereby preventing damage to structural and functional integrity of cells [71]. Earlier, [74,91] reported significant protection to chloroplast functioning, structure of thylakoids and stomata by lowering of the oxidative damage via up-regulation of the antioxidant metabolism. Up-regulation of the AsA–GSH pathway maintains the ratio of NADP/NADPH, leading to optimization of photosynthetic electron transport. Earlier, up-regulation of the antioxidant system due to drought stress has been reported by [7,92]. AMF-mediated up-regulation of the antioxidant system benefits plant metabolisms by maintaining the redox homeostasis. Earlier, [93] demonstrated up-regulation of the antioxidant metabolism due to AMF inoculation, leading to increased photosynthesis in *Robinia pseudoacacia.* In drought-stressed watermelon, AMF-mediated up-regulation of antioxidant functioning and redox homeostasis maintenance has been reported to prevent negative effects to considerable levels [74]. AsA and GSH are key components of the redox buffer and, in addition, can directly scavenge toxic ROS [94]. Plants exhibiting up-regulated expression of the antioxidants counter stress conditions better [95], therefore contributing to improved yield productivity. In addition, inoculation of AMF increased phenol content, which could have further strengthened the antioxidant system. Polyphenolic compounds act as active scavengers of free radicals, thereby protecting major cellular structures and their functioning [96]. AMF-mediated increases in phenol accumulation under drought-stress conditions were earlier reported by [9,97].

Drought tolerance via up-regulated antioxidant systems in AMF-colonized maize plants was further enhanced by greater synthesis of compatible osmolytes, including proline, glycine betaine and sugars. Plant species exhibiting higher accumulation of osmolytes show improved tolerance and growth performance under stress through maintenance of tissue water content, protein structure and functioning [94]. Similar to our results, [7] demonstrated increased accumulation of proline and sugars in wheat under drought stress. It has been reported that enhancement of the accumulation of osmolytes is regulated through modulations in their assimilatory pathways with up- and down-regulation of their synthesis and catabolism [80,98,99,100]. Therefore, it is suggested that AMF inoculation of drought-stressed plants may have improved the synthesis and down-regulated the catabolism of osmolytes, resulting in significant increases in their accumulation. AMF-mediated proline, sugars and glycine betaine accumulation may have protected the maize plants from ROS-induced oxidative damage to proteins and membranes [19]. Accumulation of osmolytes is considered a ubiquitous response for accelerating the water uptake under drought conditions [14], and AMF-mediated enhancement in their accumulation observed in the present study justifies the beneficial role of AMF in improving the growth performance of maize under water deficit conditions. Sugars are key ROS scavengers and are believed to regulate the interplay between ROS signaling and abiotic stress tolerance for facilitating plant development [101]. In drought-stressed water melon, inoculation of AMF has been reported to improve the accumulation of proline and sugars, resulting in greater tolerance and growth protection [74]. In the present study, AMF-mediated enhancement in free amino acids was correlated with increased NR activity, thereby contributing to greater accumulation of amino acids; such a correlation has also been reported earlier [3]. Improved glycine betaine accumulation has been reported to protect photosynthesis by enhancing Rubisco activity under salinity stress [102]. Therefore, it can be inferred that AMF-mediated glycine betaine accumulation in the present study may have protected Rubisco functioning, thereby optimizing photosynthesis under drought conditions, as has been reported by [74]. Compatible osmolytes serve as energy reserves for quick growth after stress release. Zhang et al. [98] have also reported increased glycine betaine accumulation in maize due to PEG-induced osmotic stress. In addition to increased osmolyte accumulation, AMF contributes to improved water retention capacity through the external hyphae [103].

## 5. Conclusions

Conclusively, inoculation of AMF (*G. versiforme*) imparted a positive influence on the growth and development of maize under drought stress by strengthening key tolerance mechanisms. AMF up-regulated the antioxidant system, leading to amelioration of oxidative damage to membrane functioning and photosynthetic performance. In addition, higher accumulation of osmolytes reflected in maintenance of tissue water, and further strengthening of the antioxidant defense system, contributed to structural and functional stability of the whole plant. Improvement in uptake and assimilation of minerals concomitant with enhancement in photosynthetic functioning favors the role of AMF in protecting maize growth and production under drought conditions. AMF-mediated holistic integration of the tolerance strategies for improving growth of maize under stressful conditions is suggested.

## Figures and Tables

**Table 1:** Effect of drought stress on shoot height (cm) and dry weight (g plant^−1^), and root AMF colonization in *Zea mays* L. Data presented is mean (± SE) of three replicates and different letters show significant difference at *p* < 0.05. MD—moderate drought, SD—severe drought, AMF—*G. versiforme*.

Treatment	Plant Height (cm)	Shoot Dry Weight (g)	Root Colonization %
Control	55.50 ± 1.23c	3.38 ± 0.36c	-
MD	45.32 ± 32d	2.61 ± 0.19d	-
SD	32.93 ± 1.31e	1.58 ± 0.06f	-
AMF	75.66 ± 1.52a	5.94 ± 0.60a	93.33 ± 4.92a
MD + AMF	68.93 ± 2.16b	4.24 ± 0.24b	67.78 ± 2.94b
SD + AMF	46.93 ± 1.32d	2.05 ± 0.17de	57.78 ± 2.94c

**Table 2:** Effect of drought stress on activity of nitrate reductase (µmol NO_2_^−^ released hr^−1^ g^−1^ FW), and content of nitrogen, phosphorus and potassium (mg g^−1^ DW) in *Zea mays* L. with and without AMF inoculation. Data presented are means (± SE) of three replicates and different letters show significant difference at *p* < 0.05. MD—moderate drought, SD—severe drought, AMF—*G. versiforme*.

Treatment	NR	N	K	P
Control	0.686 ± 0.021c	16.1 ± 0.97bc	18.12 ± 1.15c	11.55 ± 0.33
MD	0.391 ± 0.018e	13.2 ± 0.71d	15.20 ± 1.04d	9.96 ± 0.02
SD	0.201 ± 0.012f	8.11 ± 0.56f	10.50 ± 0.92e	5.23 ± 0.01
AMF	1.09 ± 0.093a	22.2 ± 2.14a	29.50 ± 2.11a	18.48 ± 0.01
MD + AMF	0.933 ± 0.060b	17.2 ± 1.68b	25.62 ± 1.97b	13.80 ± 0.32
SD + AMF	0.447 ± 0.037d	12.1 ± 1.02de	15.26 ± 1.12d	9.52 ± 0.40
